# CROSS-TISSUE EPIGENETIC AGE COMPARISONS

**DOI:** 10.1093/geroni/igae098.2289

**Published:** 2024-12-31

**Authors:** Abner Apsley, Idan Shalev

**Affiliations:** The Pennsylvania State University, University Park, Pennsylvania, United States; The Pennsylvania State University, University Park, Pennsylvania, United States

## Abstract

Accurately measuring the process of biological aging – the progressive deterioration of cells, tissues and organs – is crucial in studying the effects of aging interventions. A common group of tools used to measure the process of biological aging are epigenetic clocks (i.e., algorithms that take advantage of the age-related changes observed in the DNA methylome to estimate an individual’s biological age). Although epigenetic clocks have been trained almost exclusively using blood-based tissues, there is a growing desire to estimate epigenetic age using less-invasive mouth-based tissues (i.e., buccal or saliva). However, by definition, differentiated cell types exhibit unique DNA methylation landscapes and age-related alterations to the DNA methylome. Therefore, using epigenetic age estimates via mouth-based tissues that rely on blood-based tissues can introduce errors when estimating biological age. We tested the within-person comparability of common epigenetic clocks across five tissue types: buccal epithelial, saliva, dry blood spots, buffy coat (i.e., leukocytes), and peripheral blood mononuclear cells. Our sample included individuals from 9-70 years old (N=83) across 284 distinct tissue samples. Overall, significant differences in mouth-based versus blood-based tissue estimates were observed, with differences of more than 30 years being observed in some clocks. In addition, most blood-based clocks exhibited a low correlation with buccal and saliva tissues, even after controlling for cellular proportions, indicating that buccal and saliva are both uniquely different from blood-based estimates. Our findings indicate the deviance of mouth-based versus blood-based epigenetic clock estimates, and highlight important differences of using diverse tissue types to estimate biological age.

